# Fern botanist Ren-Chang Ching and his taxonomic system

**DOI:** 10.1007/s13238-020-00758-y

**Published:** 2020-07-10

**Authors:** Xunfeng Xu, Lei Fu

**Affiliations:** Zhejiang Normal University, Jinhua, 321004 China

**Figure 1:**
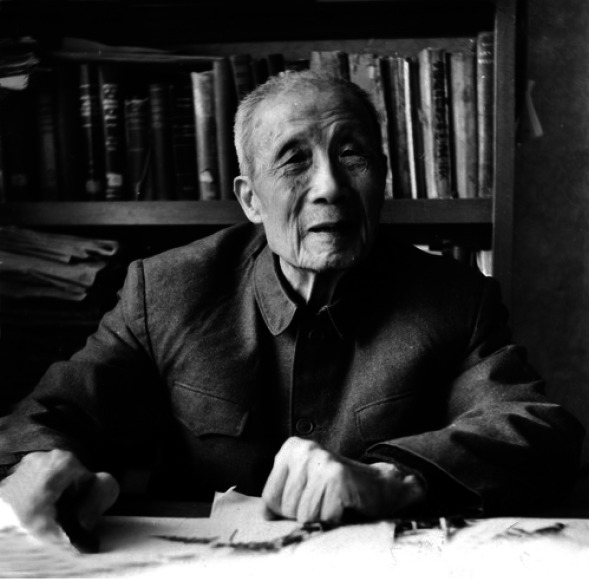
Ren-Chang Ching

Ren-Chang Ching (秦仁昌,1898–1986) is a famous botanist in China (Fig. 1). He is known as the father of Chinese pteridology, having done extensive research on ferns. The Ren-Chang Ching System established by him has made great contributions to fern taxonomy all over the world.

**Figure 2:**
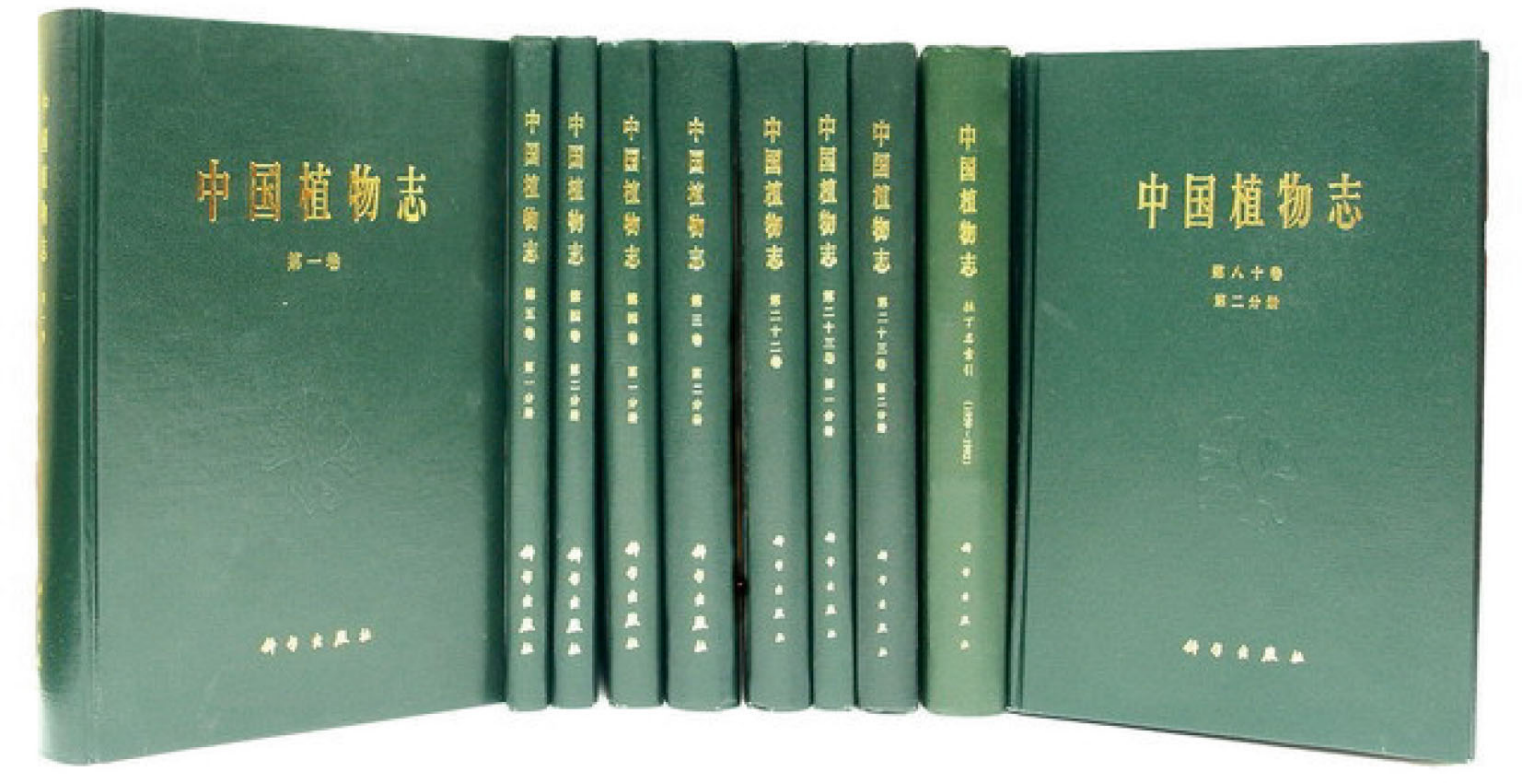
*Chinese Flora*

When he was studying in the First Agricultural School in Jiangsu Province, he became extremely interested in botany under the influence of Rong Chun (陈嵘) and Chong Shu Chien (钱崇澍). After graduation, he was admitted to University of Nanking and obtained his bachelor’s degree in forestry. He worked as an assistant at Southeast University and was employed later by the Natural History Museum of Academia Sinica. In 1929, he went to the Botany Museum of the University of Copenhagen in Denmark, and began to engage in research on fern taxonomy under the guidance of professor C. Christensen, who was one of the world’s leading authorities on ferns. While studying in Europe, he inspected Chinese fern specimens kept in various countries in detail, which laid a solid foundation for the later establishment of his fern classification system. After returning to China, he worked as a researcher at the Institute of Hydrobiology in Peiping. In 1934, he was sent to Jiangxi by Hsen Hsu Hu (胡先骕) to establish the Lushan Botanical Garden and served as its director. During the War of Resistance against Japan, he established a fern research center in Kunming. From 1945, he was appointed successively as an associate professor at the Department of Forestry and Biology of Yunnan University. In 1949, he also served as the Deputy Director of the Forestry Bureau of Yunnan Province. He was elected as a member of the Academic Department of the Chinese Academy of Sciences in 1955, and was transferred to the Institute of Botany of the Chinese Academy of Sciences in the following year, presiding over the compilation of *Chinese Flora* (Fig. 2).

Ren-Chang Ching made a great contribution to Chinese botany, especially in the field of pteridology. While working at Southeast University, he became interested in fern research with the support of Woon Young Chun (陈焕镛). When Ren-Chang Ching was a botanical technician at Academia Sinica, he realized that research on ferns in China was quite backward. To reverse this situation, it was necessary to sort out the research on ferns in China available at the time. However, research on the ferns of China has a history of 180 years in foreign countries with abundant contents, with the protologue materials and type specimens scattered abroad. Therefore, it was difficult to carry out research on ferns in China (Wu, 1986). He learned English, French, Latin and other languages by himself, and went to Europe for further studies and investigations. In order to find out the patterns of ferns of China, he took 18,300 photos of fern patterns (Wang, 1998), collected a lot of valuable information about ferns and laid a solid foundation for the fern taxonomy of China at the Royal Botanic Gardens Herbarium and British Museum. After returning to China, he revised the first draft of the *Monograph of Chinese Ferns* drafted in 1930, which was the first complete monograph of Chinese ferns. Ren-Chang Ching devoted himself to the study of botany for more than 60 years, and published over 160 articles and monographs, as well as 15 translations. He participated in organizing and compiling the *Chinese Flora*, one of the world’s largest floras, and led young people in their challenging work to complete the second volume in 1959, immediately starting the compilation of the next several volumes afterwards.

Ren-Chang Ching proposed the Ren-Chang Ching System. Prior to this, Joseph Dalton Hooker divided the true ferns into two families, *Gleicheniaceae* and *Polypodiaceae.* The *Polypodiaceae* contained almost 85% of all known ferns, but their relationships were complex, which led to a stagnation of systematic research. In 1940, Ren-Chang Ching published the *Natural Classification System of Polypodiaceae*, in which he divided plants of *Polypodiaceae* into 33 families and 249 genera, and proposed 5 evolutionary clues (Xing, 1994). His system ended the conservative classification system of Joseph Dalton Hooker and solved the biggest problem of fern botany at that time, which made great contributions to the worldwide fern taxonomy. He knew deeply that taxonomy needs to be constantly updated based on the latest morphological findings and he also conducted in-depth research on anatomy and cytology. He published the *Systematic arrangements of families and genera of Chinese pteridophytes with corresponding names in Chinese* in 1954 and *The Chinese fern families and genera*: *systematic arrangement and historical origin* in 1978, which moved the Chinese fern classification system to a new level. This system divided the fern phyla into 5 subdivisions, 3 classes, 11 orders, and 63 families, while establishing 5 new families and 8 new genera at the same time, which clarified the origin and evolution of the family (Wu, 1984). The Ren-Chang Ching system won the First Prize for Natural Science of the Chinese Academy of Sciences in 1989 and the First Prize for National Natural Science Award in 1993. The Ren-Chang Ching system is widely applied by the academic community around the world.

In addition to his own research, Ren-Chang Ching attached great importance to the training of talents. He vigorously advocated for the training of scientific and technical personnel, supported promising taxonomists to study abroad, and shared ideas with international peers. Fern botanists all over the country grew up under his guidance. He was very concerned about the juniors, treated the students as equals, and did his best to help them regardless of age and qualifications. For example, Kuo Mei Feng (冯国楣) and Wen Tsai Wang (王文采) were trained by Ren-Chang Ching. In order to cultivate domestic botanical scholars, he translated more than 800,000 words of *Latin of Botany*, the botany sections of the *Modern Dictionary of Science and Technology* and *Webster*’*s Dictionary* (Qiu and Zou, 1983). He has made great contributions to the growth of the Chinese botanical community.

Ren-Chang Ching devoted his whole life to his studies and made great contributions to the development of science in the motherland. His distinguished achievements earned him a high reputation both at home and abroad. In 1988, at the 90th anniversary of the birth of Ren-Chang Ching, the president of the International Fern Society Hennipman said, Ren-Chang Ching is not only the father of pteridology in China, but also the world.
